# Modelling Water Uptake Provides a New Perspective on Grass and Tree Coexistence

**DOI:** 10.1371/journal.pone.0144300

**Published:** 2015-12-03

**Authors:** Michael G. Mazzacavallo, Andrew Kulmatiski

**Affiliations:** 1 Department of Biological Sciences, University of Alaska, Anchorage, Alaska, United States of America; 2 Department of Wildland Resources and the Ecology Center, Utah State University, Logan,Utah, United States of America; US Geological Survey, UNITED STATES

## Abstract

Root biomass distributions have long been used to infer patterns of resource uptake. These patterns are used to understand plant growth, plant coexistence and water budgets. Root biomass, however, may be a poor indicator of resource uptake because large roots typically do not absorb water, fine roots do not absorb water from dry soils and roots of different species can be difficult to differentiate. In a sub-tropical savanna, Kruger Park, South Africa, we used a hydrologic tracer experiment to describe the abundance of active grass and tree roots across the soil profile. We then used this tracer data to parameterize a water movement model (Hydrus 1D). The model accounted for water availability and estimated grass and tree water uptake by depth over a growing season. Most root biomass was found in shallow soils (0–20 cm) and tracer data revealed that, within these shallow depths, half of active grass roots were in the top 12 cm while half of active tree roots were in the top 21 cm. However, because shallow soils provided roots with less water than deep soils (20–90 cm), the water movement model indicated that grass and tree water uptake was twice as deep as would be predicted from root biomass or tracer data alone: half of grass and tree water uptake occurred in the top 23 and 43 cm, respectively. Niche partitioning was also greater when estimated from water uptake rather than tracer uptake. Contrary to long-standing assumptions, shallow grass root distributions absorbed 32% less water than slightly deeper tree root distributions when grasses and trees were assumed to have equal water demands. Quantifying water uptake revealed deeper soil water uptake, greater niche partitioning and greater benefits of deep roots than would be estimated from root biomass or tracer uptake data alone.

## Introduction

Plant uptake of soil resources is one of the most fundamental processes of life on earth. It is believed to determine when, where and how much plants grow [1–3]. Particularly in arid and semi-arid systems, which represent nearly half of terrestrial ecosystems, plant growth is highly sensitive to soil water availability [4, 5] and plant coexistence is believed to reflect differences in plant rooting patterns [3, 6, 7]. For example, Walter’s two-layer hypothesis suggests that grasses predominate in savannas due to greater water use efficiency and dense shallow roots but that woody plants can coexist with grasses due to deep roots [1, 8]. This hypothesis was developed for drier savannas (i.e., < 500 mm precipitation), but it is often applied to a wide range of arid and semi-arid ecosystems [8]. Testing this hypothesis requires measurements of the location, timing and extent of water uptake by grasses and trees in the field, yet much of the support for the two-layer hypothesis continues to be inferred from observations of root distributions and not plant water uptake [6, 8–10].

Root biomass is fairly easy to measure but it may not be a good indicator of plant water uptake [11–13]. Large, suberized roots represent a large portion of root biomass but do not absorb soil water [11, 13]. Small, fine roots and root hairs do absorb soil water but only when soil water is plant available [14, 15]. Also, it is often difficult to differentiate the roots of different plant species [16, 17]. Natural abundance stable isotope analyses have provided valuable insight into the depth of water uptake by different species in the field [10, 18–20], but this approach typically allows only broad distinctions in water use, cannot distinguish water use below 50 cm and can be difficult to execute where precipitation events are frequent [21, 22]. Where grass and tree roots have been differentiated by visual inspection, stable isotope analyses or genetic testing, most tree roots have often been found in shallow soils suggesting that niche partitioning cannot be large enough to allow grass and tree coexistence [17, 23, 24].

Lacking measurements of root water uptake and therefore direct evidence of niche partitioning, some authors have suggested that aboveground factors alone, such as demography, fire and herbivory determine tree and grass coexistence in savannas [7, 25]. Whether aboveground or belowground processes determine savanna structure and function remains a significant question [6]. Quantifying root water uptake is a necessary step towards addressing this question [4, 10, 14] that has the added benefit of improving understanding of hydrologic cycles [26–28].

Quantifying root water uptake by different plants in the field remains a difficult task [2, 12, 14]. Here we use a hydrologic tracer experiment to measure the abundance of active grass and tree roots at different depths [27]. Because plants may absorb tracer from dry soils and because tracer uptake doesn’t reveal the amount of soil water absorbed, we use a soil water movement model to estimate the amount of water likely to flow through the active roots identified in the tracer experiment [6, 15, 29]. More specifically, our general approach was to 1) inject the hydrologic tracer deuterium oxide (D_2_O) into five soil depths, three times during a growing season in a sub-tropical savanna to determine the proportion of tracer uptake from each depth by dominant grasses and trees [12, 27], 2) parameterize Hydrus 1D, a widely-used soil water movement model [15], with tracer-derived estimates of active root distributions. This approach produced modeled estimates of the amount of water removed by grasses and trees from different soil depths over a growing season [22, 29]. Model estimates were compared to independent measurements of soil water content. Finally, to determine which rooting distribution provides more water, we use the water movement model to estimate water uptake when grasses and trees are equally abundant.

This research represents an attempt to use hydrologic tracer data in a soil water movement model to estimate species-level water uptake at our study site. Here we rely on Penman-Monteith (P-M) models to estimate evapotranspiration (ET) though these models were not designed or validated for use in aerodynamically heterogeneous communities. While we validate estimates of plant water uptake using soil water content data and constrain ET estimates to match independent estimates of ET from a nearby eddy-covariance flux tower, we did not validate independent components of the P-M model. Before this approach can be applied broadly, critical components of this modeling approach, namely aerodynamic resistances and preferential flow paths in the soil will need to be validated.

## Methods

Research was conducted in a sub-tropical savanna on shallow clay soils during the 2009/2010 growing season, Letaba, Kruger National Park, South Africa (-23.429° S, 31.053° E; 200 m elevation). This research was approved by South African National Parks, registration number 213896412. Soils are shallow (50–100 cm) red and dark clays derived from the underlying basaltic bedrock [30]. Mean annual precipitation is 450 mm and 521 mm fell during the study period, which included a notable mid-season drought (S1 and S2 Figs). The ‘mopaniveld’ vegetation is dominated by the tree *Colophospermum mopane* and the C4 grass *Bothrichloa radicans*. Woody plant crown cover was 20 ± 3%. The Mopaniveld is described as a water-limited ecosystem that covers about 555,000 km^2^ of southern Africa[30, 31]. *C*. *mopane* can grow to 15 m tall [32, 33], but was on average about 4 m at our site. Other woody plants included *Combretum apiculatum* and *Flueggia virosa* but these plants represented less than 5% total vegetative cover. The perennial grass *Bothrichloa radicans* dominated vegetative cover. In descending order of abundance, the following perennial grasses were also common: *Urochloa mosambicensis*, *Panicum maximum*, *Aristida transvaalensis*, *Schmidtia pappophoroides* and *Digitara eriantha*.

### Hydrological tracer experiment and root biomass

A depth-controlled tracer technique was used to assess the relative abundance of active roots by depth and plant species [21]. Briefly, 48 10-m^2^ circular plots were placed 30 m apart in a grid in a roughly 3.5 ha area. Three replicate plots were assigned to each depth (5, 10, 20, 30 and 70 cm) by month (December, February, April) treatment combination and the remaining three plots were controls. On the assigned tracer addition date, 1 ml of D_2_O (70% deuterium, 30% hydrogen; Cambridge Isotopes, MA, USA) followed by 2 ml tap water was injected into each of 444 pilot holes drilled in a 15 cm x 15 cm grid to the target depth in each plot resulting in a total of 19,980 injection points over the season. Each 10 m^2^ plot therefore received 1.3 L or 0.13 mm of tracer water. This represented <10% of typical daily evapotranspiration (ET) at the study site [27]. One to three days following tracer injection, non-transpiring tissues [21, 34] from grasses and trees were clipped, placed in sealed glass sample tubes and kept on ice until sample extraction (within two weeks). Sample timing was deduced from earlier research which demonstrated peak tracer concentrations in plant tissues one to two days following injection [12, 21]. Samples of common species were composited to produce 3–5 sub-replicate samples for each species. During each sampling period, five to eight samples were removed for each target species from a control plot. Twenty-two species were sampled, though *C*. *mopane* (48%), *B*. *radicans* (22%), *A*. *transvaalensis* (13%), *P*.*maxicum* (2%), *S*. *pappophoroides* (2%) and *U*. *mosambicensis* (2%) and a mixture of forbs (7%) represented 94% of samples.

Soil cores were taken in each plot 2 to 3 days after injections. Samples from these cores were used to measure root biomass, soil water content, soil water potential and isotope concentrations. Soil subsamples were removed in 20 cm increments for most of the soil core but in 10 cm increments above and below target injection depths (S3 Fig). Sampling was performed by a team of four to five people to reduce sampler error [35]. For root biomass analyses, subsamples were dried and sieved through a 2 mm sieve. Coarse roots (i.e., above the sieve) and fine roots (i.e., below the sieve) were collected and weighed together. For isotope analyses, plant and soil samples were extracted using a batch cryogenic distillation procedure [36]. Samples with a noticeable aroma or cloudy appearance were extracted with activated carbon. Extracted water samples were pipetted into 2 ml vials, shipped to the University of Alaska Anchorage’s Environment and Natural Resources Institute Isotope Laboratory and analyzed for hydrogen and oxygen stable isotope ratios on a wavelength scanned cavity ring-down spectrometer (L1102-I Cavity ring-down spectrometer; Picarro Instruments, CA, USA). Isotope values of water extracted from plant and soil samples were reported in delta notation (δ) and converted to deuterium excess values (δ_e_) to control for natural enrichment as follows: δ_e_ = δD–[(8 * δ^18^O) + 10][37].

To account for tracer dilution differences associated with different-sized plants and rooting zones, δ_e_ values were converted to proportional tracer uptake as a function of soil depth as follows: $\frac{S_{n}-C}{\sum_{n=1}^{j}(S_{n}-C)},$ where *S*_n_ is the mean δ_e_ value of samples from treatment level *n* (e.g., grasses at 5 cm depth in the first replicate plot in December), and *C* is the mean δ_e_ value of control samples for that functional group [21, 38]. In the denominator, values (i.e., *S*_*n*_ *−C*) were summed across all depths from 1 to *j* (i.e., 5–70 cm). This value was calculated for each plant functional type (i.e., grass or tree) in a plot, producing three replicate proportion values for each plant type x depth x date combination. Variation in tracer uptake among replicate plots was assumed to reflect variation in rooting distributions on the landscape and is the only source of reported error.

### Calculating species- and depth-specific ET

Tracer data provided estimates of the distribution of active grass and tree roots with depth. To account for the presence of dry soils at different depths or times of year we used a soil water movement model (Hydrus 1D). Hydrus 1D simulates water flow through the soil matrix, evaporation and water uptake from a single root distribution. Because Hydrus 1D simulates a single root distribution, it was necessary to 1) combine tracer data from different plant types to produce a single root distribution that was used as a Hydrus 1D input, then 2) parse Hydrus 1D model output of water uptake by all plant roots into grass and tree components.

To produce a single root distribution from species-level tracer uptake data, tracer data were weighted by grass and tree ET (calculations described below). It would not be appropriate to use the average of grass and tree rooting distributions because grass cover on the landscape is much greater than tree cover. It also may not be appropriate to weight grass and tree root distributions by leaf area since one unit of grass leaf area may transpire more or less than one unit of tree leaf area. To provide an example, our ET models may estimate that total ET during January was 30 mm. Multiplying this estimate by the proportion of grass and tree tracer uptake by depth may reveal that grasses and trees transpired 8 and 2 mm from the 0–5 cm depths. In this case the total root distribution used as input for Hydrus 1D was estimated to have 10 of 30 mm uptake or 33% of active roots in the 0–5 cm depths. This estimate of water uptake assumes that water is always available at all soil depths. Hydrus 1D outputs of total root water uptake accounted for dry soils but needed to be parsed to grass and tree components. We used the same ET-based weightings used to create a single root distribution for model input to parse model output. For example, due to dry surface soils, Hydrus 1D may estimate that only 5 mm of soil water was absorbed by roots in the 0–5 cm depth in January. In this example, we would assume that 80% of this total root water uptake was performed by grasses because grasses and trees were estimated (under fully wet conditions) to transpire 8 and 2 mm of water from the top 5 cm, respectively.

As a measure of inherent competitive ability, we were also interested in estimating how much water grasses and trees absorbed when both were equally abundant. To do this, we created a single root distribution as Hydrus 1D input by simply averaging the proportion of tracer uptake by depth for grasses and trees. Parameterized in this way, Hydrus 1D estimated total root water uptake by depth assuming grasses and trees had equal transpiration demands. To parse total Hydrus 1D output into grass and tree components, total root water uptake was multiplied by the proportion of grass and tree tracer uptake at each depth.

### Evapotranspiration models

ET was calculated for the four dominant grasses (*A*. *transvaalensis*, *B*. *radicans*, *P*.*maxicum*, *and U*. *mosambicensis*) using the standard FAO Penman-Monteith (P-M) model [39] and a similar model modified for use with trees was used for *C*. *mopane* [40]. These five species represented 87% of total leaf area. For each species, species-specific stomatal conductance and plant height data were used to parameterize the P-M model. Because the P-M model was parameterized to work with leaf area index values of 1.0 or more, ET was calculated for each species assuming a leaf area index of 1.0 [22]. We then multiplied this ET estimate by observed leaf area of the target species [22]. In other words, we assumed that a plant with 10% ground cover transpired 10% as much as a plant with 100% ground cover [22].

Grass ET was estimated using the standard FAO P-M model calculated on an hourly basis[40] as follows:

$$\lambda ET=\frac{\Delta \left(R_{n}-G\right)+\;\rho_{a}c_{p}\frac{\left(e_{s}-e_{a}\right)}{r_{a}}}{\Delta +\gamma \left(1+\frac{r_{s}}{r_{a}}\right)}$$

where ET is expressed as mm hour^-1^, λ is the latent heat of vaporization assumed to be 2.45 MJ kg^-1^, R_n_ is net radiation at the vegetation surface (MJ m^-2^ hour^-1^), G is soil heat flux density (MJ m^-2^ hour^-1^), e_s_ is the saturation water vapor pressure, e_a_ is the actual vapor pressure, (e_s_-e_a_) is the vapor pressure deficit of the air (kPa), ρ_a_ is the mean air density at constant pressure (1.293 kg m^-3^), c_p_ is the specific heat of the air at constant pressure (1.013 10^−3^ MJ kg^-1^°C^-1^), Δ is the slope of saturation vapor pressure versus temperature curve (kPa°C^-1^), γ is psychrometric constant (0.067 kPa°C^−1^), r_s_ is bulk surface resistance (s m^-1^), and r_a_ is bulk aerodynamic resistance (s m^-1^). Surface resistance is calculated as a function of plant leaf area and stomatal resistance [39, 40]. Aerodynamic resistance is estimated from plant height and wind speed [39, 40]. This P-M model is essentially an energy budget model that partitions the energy in incoming solar radiation among outgoing long-wave radiation, sensible heat in the air and soil and latent heat.

The PM model should have produced reasonable estimates of ET for the continuous grass canopy, but would not be appropriate for modelling ET in the discontinuous tree canopy. The ET model we used for trees was developed and validated using discontinuous tree canopies in orchards[41–43]. Tree ET was calculated using the following modified version of the P-M equation[40–43]:

$$ET=\frac{\Delta R_{n}+\;\eta \rho_{\alpha }c_{p}\left(e_{s}-e_{a}\right)g_{a}}{\lambda \left[\Delta +\;\eta \gamma \left(\zeta +\;\frac{g_{a}}{g_{s}}\right)\right]}$$

Where g_a_ is the boundary layer conductance, g_s_ stomatal conductance, η is a factor that represents the ratio between the boundary layer conductance for water vapor and for sensible heat at constant pressure, and ζ is equal to 2, which represents stomata located on one side of the leaf. Leaf boundary layer conductance (g_a_, in m s^-1^) was estimated using a commonly used model for trees proposed by Landsberg and Powell[41] but see[42], which accounts for the mutual sheltering of clustered leaves as:

$$g_{a}=\;0.0172p^{-0.56}{\left(U/D\right)}^{0.5}$$

Where D is the characteristic leaf dimension (m), and U is average wind speed (m s^-1^) measured at mid-canopy height. The parameter p is the ratio between the tree leaf area and the area projected onto a vertical plane.

Stomatal conductance was modified for the modified (i.e., tree form) P-M equation as follows:

$$g_{s}=\;g_{max}\left(\frac{1-\;\delta D_{a}}{1+\;\beta Q_{p}^{-1}}\right)$$

Where β and δ are empirical coefficients [43], *g*_*max*_ is the maximum stomata conductance determined with porometer measurements, D_a_ is the ambient vapor pressure deficit, and is the photosynthetic photon flux density on the leaf surface[44].

Stomatal conductance measurements were needed to estimate both *r*_*s*_ and g_max_ and were measured on focal species throughout the day on 8 dates from December to April using solid-state leaf porometers (SC-1; Decagon Devices, Pullman, WA, USA). To control for effects of changing environmental conditions, conductance measurements were made from six dominant target species every 15 minutes. Tree readings were taken from the top, middle, and lower portions of the canopy. Adaxial conductance was not observed on tree leaves, but was measured on grasses. Conductance datasets were described with a best-fit polynomial curve to estimate hourly conductance throughout the season[22].

Plant height, leaf area, projected leaf area and wind speeds were needed to estimate *r*_*a*_. Plant heights were measured on 30 randomly-selected plants of each target species along a transect during each sampling campaign. Leaf area was measured in 16 randomly selected 1 m^2^ plots during each of six sampling periods. Plumb lines were used to indicate plot locations within tree canopies. Clipped vegetation was frozen within six hours of sampling and scanned on a portable leaf area scanner (CID Inc., Camas, WA, USA). Projected leaf area was measured as leaf presence or absence in 500 points using a periscope-style densitometer with cross-hairs. Sampling points were located every m along five 100-m long transects that were separated by 30 m. Wind speed was measured at grass canopy height (0.3 m) and mid tree-canopy height (2 m).

### Hydrologic model

Proportional tracer uptake values were used to parameterize the hydrologic model, Hydrus 1D [15]. This model simulates water flow through the soil and roots and provides estimates of soil water content and root water uptake by depth as a function of climate, soil and plant traits. The van Genuchten-Mualem hydraulic model was used with no hysteresis in the retention curves. Water flow parameters were derived from measurements of soil texture, observed maximum and minimum soil water contents and associated water potentials, and assumed bulk densities of 0.8, 1.0, 1.1, 1.2, and 1.3 g cm^-3^ for the 5, 10, 20, 30 and 70 cm depths, respectively. These bulk densities were inferred from personal observation, were consistent with estimates derived from soil texture-derived estimates [45], and were similar to previously reported values [46] but were different from results in another study that did not observe increasing bulk density with depth [47]. Pooling of water at the soil surface was not observed in the field and a low surface bulk density was needed to prevent surface water accumulation in the model. This low surface bulk density was consistent with the presence of some leaf litter, loose soil and worm castings at the soil surface. Hydraulic conductivity (Ks) was not measured, so the inverse solution was used on data from the first month of the field season to select Ks values [15]. These Ks values were used for the remainder of the season. Upper and lower boundary conditions were defined as the atmosphere or a surface layer of water up to 2 cm deep and free drainage into subtending soils, respectively. An ‘S-Shape’ water uptake reduction model was used. In this submodel, the soil water potential at which root water uptake was reduced by 50% (i.e., the P50 value) was assumed to be -1.5 MPa. This value was chosen because leaf water potentials at the site were commonly -2.5 MPa and stomatal control was not obvious in leaf conductance measurements even in this dry range of leaf water potentials (Kulmatiski pers. obs). The exponent in the S-shaped root water uptake stress response function (i.e., the P3 parameter) was set to the recommended value of -3. The critical stress index value determines the extent to which plants can compensate for low water availability at some depths. A small value allows plants to compensate fully for dry soil layers, effectively eliminating the effect of rooting distributions, so a large value of 0.8 was used. The hCritA parameter sets a minimum pressure head at the surface beyond which evaporation is limited: a recommended value of -5MPa for fine-textured soils was used. Consistent with observed heights, plant height was assumed to be 50 cm at the beginning of the season and 100 cm for the rest of the season.

The P-M model is used to estimate ET by Hydrus 1D. The P-M model was developed for well-watered crops and as a result tends to overestimate ET in non-irrigated wildland systems[48, 49]. A ‘crop stress’ value is often used to modify the P-M model for use in wildland systems[42] but this is not an option in the Hydrus 1D model. To address this, we modified albedo to 0.6 to produce ET estimates similar to the Malopeni gas flux exchange tower located 40 km west of the study site [50]. We were forced to adjust albedo when using the Hydrus model because there was no possibility in this model to account for stomatal control and because plant water stress variables in the model did not sufficiently suppress water uptake. An interception constant value of 2 mm was used because we never observed an increase in shallow soil moisture after 2 mm precipitation events.

Meteorological data used by Hydrus 1D and P-M models were recorded on site. Wind speed (014A cup anemotmeter; MetOne, Grants Pass, OR, USA), temperature and relative humidity (Campbell Scientific CS215), solar radiation (Apogee Instruments SP-110, Logan, UT, USA), and precipitation (Texas Instruments TE-525, TX, USA) were measured hourly at both grass (0.3 m) and tree height at mid canopy (2 m) and recorded on a Campbell Scientific CR-1000 datalogger (Campbell Scientific, Logan, UT, USA).

Hydrus 1D provides depth-specific estimates of soil water content over time. Model estimates of soil water content were compared to independent estimates of soil moisture that were measured at 10 soil depths between 5 and 170 cm using soil water potential sensors (Campbell Scientific 229 sensors, Logan, UT, USA). Prior to installation, each sensor was calibrated using an endpoint test and by taking measurements in soils from one of three appropriate depth strata (0–30, 30–60, or 60–90 cm) that were equilibrated to each of five known water potentials for 16 h [51]. Water potentials of the equilibrated soils were determined using the chilled-mirror technique (WP4T water potential meter; Decagon Devices, Pullman, WA, USA). Sensors were placed in pilot holes established in the undisturbed wall of a soil pit located at the site where micrometeorological measurements were made. A loop of sensor cable was placed below the sensor to prevent the creation of a preferential flow path to the sensor. Calibrated sensors were placed at 5, 10, 20, 30, 40, 50, 75, 100, 160 and 170 cm in the soil profile. Following sensor installation, the soil pit was filled and compacted and grass root mats were replaced. Soil water potentials were converted to gravimetric soil moisture using published soil characteristic curves [52].

In all cases, values of water uptake per depth strata were converted to a per cm basis by dividing by the depth increment in the strata (i.e., 20 mm of water uptake from the 20–30 cm depth strata was reported as 2 mm per cm in this strata). This allowed estimates of the depth at which 50% of root biomass, tracer uptake or water uptake occurred. Values are reported as a running average of 15 cm increments to smooth their distribution with depth.

### Statistical Analyses

To test for a general pattern in tracer uptake by grasses and trees across the growing season, we performed a three-way randomized factorial analysis for the effects of month, plant type and depth on proportion uptake across the season. A generalized linear mixed model and logit link was computed using the GLIMMIX procedures in SAS/STAT for Windows, Release 9.3 (SAS Institute Inc., Cary, North Carolina; Appendix).

### Niche overlap

Niche overlap was calculated using the proportion of tracer uptake and measures of plant available water (PAW) using EcoSim ver. 7 [12, 53]. Pianka’s standardized overlap value: $O_{jk}=\frac{\sum e_{ij}e_{ik}}{\sqrt{\sum e_{ij}^{2}e_{ik}^{2}}},$ where *O*_*jk*_ is a measure of overlap between species *j* and *k*, *e*_*ij*_ or the electivity index = *p*_*ij /*_ *R*_*j*_, where *p*_*ij*_ is the proportion that resource *i* is of the total resource used by species *j*, *p*_*ik*_ is the proportion that resource *i* is of the total resources used by species *k*, and *Rj* is a measure of the availability of resource state *j* was used [54]. This unitless measure ranges from 0 to 1, where 0 indicates that no resources are used in common (i.e., complete niche partitioning). To determine if observed overlap values were likely to result by chance, the species utilization matrices were compared to predictions from a randomized null model. Randomization algorithm three in EcoSim ver. 7, in which niche breadth is retained and zero states are reshuffled, was used because niche breadth did appear to differ by species, zero uptake did not appear to be a fixed species trait for any depth (i.e., all plants accessed some tracer from every depth sampled during one time period or another), and this approach is usually superior in detecting non-random overlap [55]. In this experiment, zero states would be depths from which a plant does not access soil water. To estimate *Rj*, we used root uptake values by depth estimated by Hydrus 1D [53]. Random niche overlap values were derived from 1000 Monte Carlo permutations of the data matrix. Observed overlap values were then compared to the distributions of the randomized values. A *P* value of < 0.05 indicates that observed niche overlap values were greater than or less than niche overlap values produced by the randomized model.

## Results and Discussion

A total of 1040 plant and soil samples were analyzed for isotope ratios: 878 experimental plant samples, 31 control plant samples, 100 experimental soil samples, and 31 control soil samples. None of the control samples demonstrated δ_e_ values two SDs above the control mean for grasses and trees, -20.82 ± 13.25 SD and -37.76 ± 11.26 SD, respectively. This suggested that sample contamination was not a problem. In contrast, 84% of plant samples from experimental plots demonstrated δ_e_ values greater than two SDs above the control mean (i.e., were considered to have received tracer [21]). Soil samples taken following tracer injection showed clear differences in tracer concentration with depth among the treatments indicating that injections placed tracers in the targeted soil depths (S3 Fig)[21, 22].

**Fig 1:**
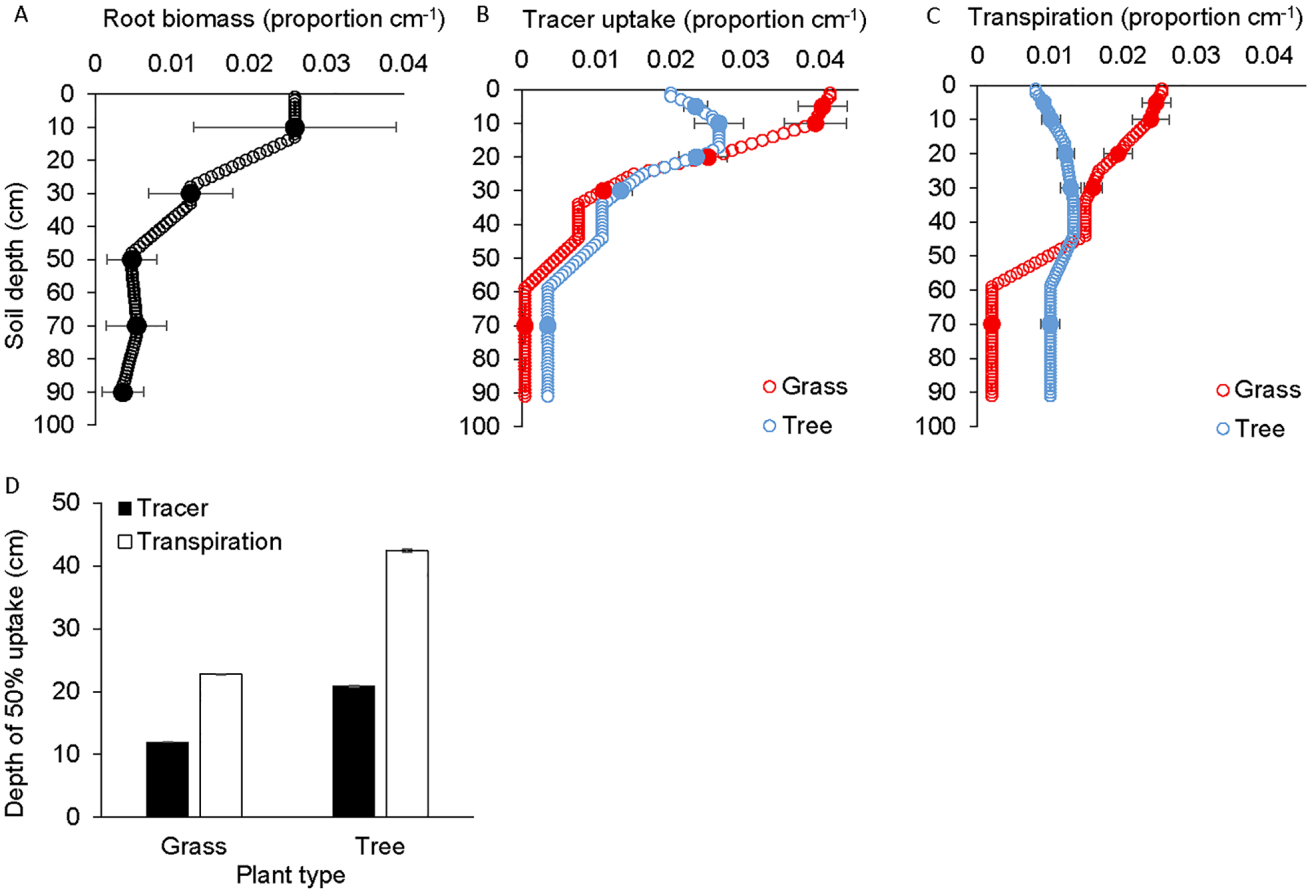
Root biomass, tracer uptake and modeled water uptake. The proportion of root biomass (A), tracer uptake (B) and modeled water uptake (C) by depth and the depth from which 50% of tracer or modeled water uptake occurred for grasses and trees (D). Tracer uptake by depth (B) reflects the relative abundance of active plant roots and shows that most plant roots are found in shallow soil. When these tracer uptake values are used in a soil water model that accounts for water availability and transpiration demand, the proportion of water uptake by depth (C) was estimated to be twice as deep as suggested by tracer uptake (B). In panels a-c the mean values (± 1SE) are shown for target depths. Remaining values (without SE bars) are interpolated and represent running averages (15-cm increments).

The distribution of root biomass indicated that half of root biomass occurred in the top 20 cm (Fig 1a). Tracer uptake differed by depth and plant type (S1 Table). Half of tracer uptake by grasses and trees occurred in the top 12 and 21 cm, respectively (Fig 1b and 1c). Most tracer uptake, therefore, occurred in shallow soils. Grasses, however, absorbed a greater proportion of tracer than trees from the shallowest pulsed soils (i.e., 5 cm) and trees absorbed a greater proportion of tracer than grasses from the deepest pulsed soils (i.e., 70 cm; Fig 1a; S1 Table, S4 Fig). These patterns of root biomass and tracer uptake were similar to grass and tree root distributions in a nearby site where 50% of grass and tree root biomass occurred in the top 9 and 12 cm, respectively [16, 27]. Results were also similar to semi-arid sites from around the world that demonstrate 50% of total root biomass in the top 7 to 28 cm [23, 56]. Tracer uptake depths were also surprisingly similar to results obtained in a more mesic, sandy site in Kruger Park that receives 750 mm precipitation [12]. Together, these results suggest that active root distributions are similar across a wide range of precipitation regimes, soil types and plant species [12, 21, 56]. These results also suggested that root biomass distributions were a good indicator of active grass root distributions. While the tracer experiment produced root distribution patterns that were similar to root biomass distributions in many sites around the world, tracer data also distinguished active from inactive roots and grass from tree roots.

Tracer uptake data indicated the location and relative abundance of active plant roots but not the amount of soil water absorbed by those roots [6, 28]. To estimate the amount of water absorbed, we used the Hydrus 1D soil water model [15]. Model results suggested that 50% of water uptake by grasses and trees occurred in the top 23 and 43 cm, respectively (Fig 1b and 1c). In both cases the depth from which the model estimated that plants obtained half of their annual water was roughly twice as deep as that estimated from tracer uptake alone. This occurred because deep soils provided a larger, more stable soil water pool (S5 Fig). More specifically, the soil water model estimated that grasses and tress together removed 104 and 148 mm of water from shallow (i.e., 0–20 cm) and deep (i.e., 20–90 cm) soils, respectively. It is important to note that Hydrus 1D model results are deterministic and not associated with any error. All error in Fig 1 reflects variation in tracer uptake among replicate experimental plots in the tracer experiment.

Results had important implications for niche partitioning. Niche overlap calculated from tracer data was very large (0.95) and significantly greater than the null model prediction (0.52, *P* < 0.001). However, when calculated from modeled estimates of root water uptake, niche overlap was not as large (0.82) and not significantly greater than the null model prediction (0.79, *P* = 0.06). This occurred because deeper soils stored more soil water and as a result small differences between grasses and trees in deep tracer uptake resulted in large differences in modeled root water uptake (Fig 1). Therefore, estimates of water uptake that accounted for soil water availability revealed patterns of niche partitioning that were not apparent from root distributions alone.

It has long been thought that grasses may be superior competitors for soil water, particularly in more arid sites where precipitation events are often small, because dense root mats provide grasses with access to soil water as it enters the soil [1, 7, 8, 57]. Here we found that when they were assumed to be equally abundant, grasses and trees were estimated to remove 102 and 149 mm of soil water over the year, respectively. In other words, relatively shallow root distributions were estimated to provide grasses with 32% less water than slightly deeper tree root distributions. This occurred because even in this fairly xeric savanna, many precipitation events were large enough to ‘push’ soil water below the small, shallow soil pool in which most grass roots were located. That water infiltrated below the majority of grass roots in this fairly xeric site with clay soils and that grass and tree rooting patterns were similar in a mesic, sandy savanna site [12], suggests that tree rooting distributions are likely to provide trees with access to more soil water than grasses in many savanna systems.

Tree root distributions were estimated to provide trees with more water than grass root distributions, yet grass leaf area (0.47 ± 0.05 m^2^ m^-2^) was more than twice tree leaf area (0.20 ± 0.01 m^2^ m^-2^; S6 Fig). As a result, across the landscape, grasses were estimated to transpire more than trees (i.e., 130 vs. 122 mm). This amount of tree transpiration (122 mm) represented 23% of precipitation and was intermediate between estimates derived from sap flux measurements in nearby sites [57, 58]. Because tree root distributions were estimated to provide more water, but grasses demonstrated more leaf area, our results support other studies that suggest that greater water use efficiency, greater access to shallow soil nutrient pools or non-equilibrium processes (e.g., fire) but not superior access to soil water explains greater grass abundance in savannas [7, 16].

While grasses have long been thought to rely on shallow soil water, here we quantify that reliance: our water model estimated that grasses obtained half of their water from the top 23 cm of soil. This is important from a climate change perspective because warmer temperatures are expected to result in fewer, larger precipitation events [59–61]. These larger precipitation events are likely to ‘push’ water deeper into the soil profile. Our results indicate that precipitation events that ‘push’ water below 23 cm at the study site are likely to increase tree relative to grass growth. This prediction is consistent with results from a precipitation manipulation experiment in a nearby site [27] and suggest that climate change and niche partitioning may provide an additional explanation for the woody plant encroachment observed in many parts of the world in the past several decades [62].

Results relied on a number of simplifying assumptions and models, yet provided reasonable estimates of water dynamics. The P-M model was used by Hydrus 1D and was used to create a single root distribution from tracer data. The P-M model is widely used in hydrological and agricultural applications and is appealing for use in species-specific modeling because it includes parameters for stomatal conductance and aerodynamic resistance. However, this model was developed and tested for dense, well-watered monoculture crops. As a result, our use of the P-M model was outside of common applications and the model overestimated ET and had to be down-weighted to provide estimates that were consistent with independent flux-tower measurements. This was more of a concern in the Hydrus 1D application where the model was being used to quantitatively predict water flow. Model estimates of ET were forced to be consistent with independent measures of ET by increasing albedo, but future efforts are needed to improve estimation of ET in natural, mixed species communities. Our P-M-derived values also overestimated ET, but because we used these values to determine the relative and not absolute amount of grass and tree ET, our use of the P-M model was buffered from the effects of overestimation. It is likely that future modeling efforts that use ET models that are better suited to water-limited, natural plant communities (i.e., with mixed height canopies and variable aerodynamic resistances) will improve model predictions [48].

Another potential source of error was that Hydrus 1D simulates only one root distribution. Hydrus 1D is widely used and tested as a soil water flow model, but the use of a water flow model that can distinguish root water uptake of multiple plant species is likely to improve model predictions [6, 63, 64]. For example, evaporative demand, sapflux rates, and different plant traits such as aquaporin density and root water potentials, are likely to result in different water flow rates from soils to roots, but these factors were not considered in this study. Finally, sensitivities in the P-M and Hydrus 1D models are fairly well understood [15, 29] but we did not perform sensitivity analyses of our integrated approach. Before our integrated approach can be reliably applied over large-scale areas, future studies should test the approach in new sites and determine the sensitivity of results to variation in factors such as preferential flow paths, soil bulk density, different root distributions, spatial distribution of trees and grasses and differences in water uptake by monoculture vs. mixed root systems.

**Fig 2:**
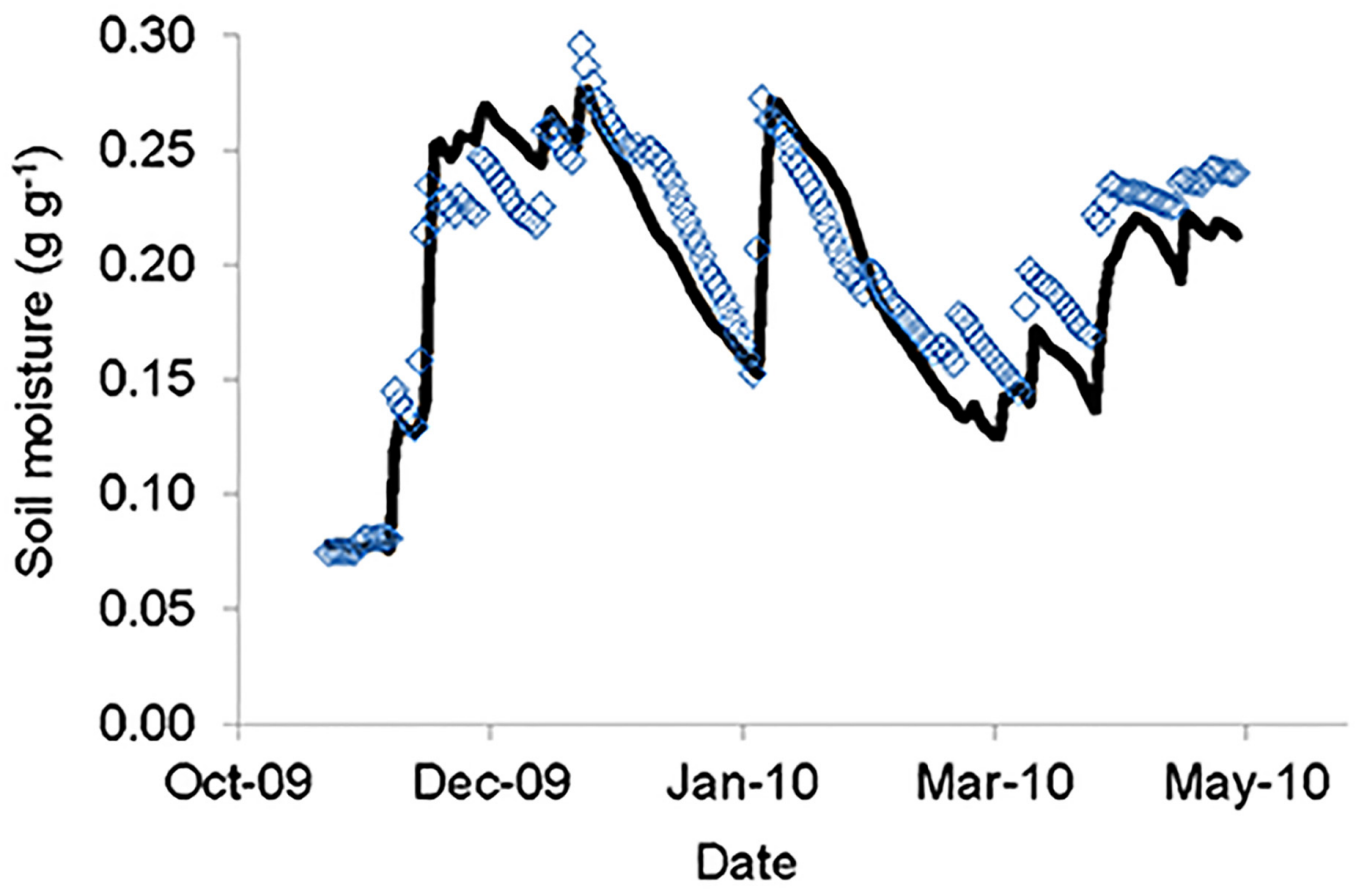
Observed and predicted soil moisture (g g^-1^) across the soil profile. Soil moisture content was observed using continuous measurements of volumetric water content and soil water potential in one soil pit at the study site. Soil water content was predicted using the Hydrus 1D soil water model parameterized with tracer-derived estimates of plant root distributions (R^2^ = 0.84). Observed and predicted values for target depths shown in S5 Fig.

Despite model assumptions, predicted soil water content was well correlated with observed soil water content across the soil profile (R^2^ = 0.84; Fig 2) and at specific depths (i.e., R^2^ values ranged from 0.59 to 0.80 for different soil depths; S5 Fig). Further, our tree ET estimates were consistent with sapflux measurements in nearby sites [50, 57, 58]. Therefore, important model parameters and outputs were consistent with several independent measures. It should be noted, however, that while predictions of soil moisture were broadly similar to observations across the season, predictions for any particular hour or day were sometimes widely different than observed values. This typically reflected a time lag in model predictions that is likely to be caused by a lack of preferential flow path water movement in the soil water model.

This research provides an example of how it may be possible to produce quantitative estimates of water use by plant species in mixed communities in the field. This type of modeling approach is needed because soil moisture data are quite rare yet critical for understanding biosphere-atmosphere feedbacks under changing climate conditions [28]. Results from this study suggest that this is a promising approach, though significant research remains to be done to determine whether or not this approach will be effective in other sites and which variables are most important to understanding water cycling and competition in mixed plant communities. In this study, quantifying water uptake, rather than inferring water uptake patterns from root distributions suggested that 1) water uptake is deeper, 2) niche partitioning is greater and 3) deeper roots provide more water than would be estimated from measures of root biomass or tracer uptake alone.

## Supporting Information

S1 Fig Mean (dotted observed) and observed (broken line) precipitation from January 2009 to December 2010, Letaba, Kruger National Park, South Africa. Gap in solid line reflects period when observed data were not available.
(DOCX)

S2 Fig Mean weekly soil water potentials from 10, 20, 30, and 70 cm depths over the 2009–2010 growing season, Letaba, Kruger National Park, South Africa. Arrows indicate the time of pulsing events.
(DOCX)

S3 Fig Deuterium concentration [delta notation in parts per thousand (‰)] of extracted soil water from target depths one day following tracer injection. (DOCX)

S4 Fig Proportional tracer uptake by grasses and trees by depth in December (a), February (b), April (c) during the 2009/2010 growing season, Letaba, Kruger National Park, South Africa. Lower case letters indicate differences among depths for grasses. Upper case letters indicate differences among depths for trees. Asterisks indicates differences between grasses and trees at a depth. Significance was determined when P < 0.05.
(DOCX)

S5 Fig Predicted (blue circles) and observed (black circles) gravimetric soil water content (θ) at (a) 5, (b) 10, (c) 20, (d) 30, (e) 70 and (f) 0–90 cm. Letaba, Kruger National Park, South Africa over the 2009–2010 growing season. predicted using tracer-derived estimates of root activity to parameterize the Hydrus 1D soil water model. The R^2^ values were 0.69, 0.59, 0.72, 0.80, 0.80 and 0.84 for panels a-f, respectively. A gravimetric water content of 0.12 is associated with a water potential of -2.5 MPa (i.e., plant unavailable water).
(DOCX)

S6 Fig (a) Leaf area and (b) mean stomatal conductance (g) for grasses and trees through the 2009–2010 growing season. Note that grass leaf area reported here was doubled when estimating transpiration. Across the growing season, grass leaf area was greater than tree leaf area (F_3, 63_ = 9.347, P = 0.002) and stomatal conductance was smaller for grasses than trees (F_3,1596_ = 22.75, P < 0.001).
(DOCX)

S1 Table Mean δD excess values (‰) ± 1 SE in plant materials sampled from plots that had received tracer at the indicated soil depths (e.g., 5 cm) and time of year (e.g., December). Values in a row followed by a different lower case letter are different at the 0.05 level.
(DOCX)
