# Supplementary material for: Modelling Water Uptake Provides a New Perspective on Grass and Tree Coexistence
Source: PLoS One. 2015 Dec 3;10(12):e0144300. doi: 10.1371/journal.pone.0144300 (PMC4669088; doi:10.1371/journal.pone.0144300)
Supplement: S1 Fig — Gap in solid line reflects period when observed data were not available. (DOCX) [file pone.0144300.s001.docx]

***S1 Figure.***  *Mean (dotted observed) and observed (broken line) precipitation from January 2009 to December 2010, Letaba, Kruger National Park, South Africa. Gap in solid line reflects period when observed data were not available.*
