# Supplementary material for: Modelling Water Uptake Provides a New Perspective on Grass and Tree Coexistence
Source: PLoS One. 2015 Dec 3;10(12):e0144300. doi: 10.1371/journal.pone.0144300 (PMC4669088; doi:10.1371/journal.pone.0144300)
Supplement: S2 Fig — Arrows indicate the time of pulsing events. (DOCX) [file pone.0144300.s002.docx]

***S2 Figure.*** *Mean weekly soil water potentials from 10, 20, 30, and 70 cm depths over the 2009-2010 growing season, Letaba, Kruger National Park, South Africa. Arrows indicate the time of pulsing events.*
