# Supplementary material for: Modelling Water Uptake Provides a New Perspective on Grass and Tree Coexistence
Source: PLoS One. 2015 Dec 3;10(12):e0144300. doi: 10.1371/journal.pone.0144300 (PMC4669088; doi:10.1371/journal.pone.0144300)
Supplement: S3 Fig — (DOCX) [file pone.0144300.s003.docx]

***S3 Figure.*** *Deuterium concentration [delta notation in parts per thousand (‰)] of extracted soil water from target depths one day following tracer injection.*
