# Supplementary material for: Modelling Water Uptake Provides a New Perspective on Grass and Tree Coexistence
Source: PLoS One. 2015 Dec 3;10(12):e0144300. doi: 10.1371/journal.pone.0144300 (PMC4669088; doi:10.1371/journal.pone.0144300)
Supplement: S4 Fig — Lower case letters indicate differences among depths for grasses. Upper case letters indicate differences among depths for trees. Asterisks indicates differences between grasses and trees at a depth. Significance was determined when P < 0.05. (DOCX) [file pone.0144300.s004.docx]

***S4 Figure.*** *Proportional tracer uptake by grasses and trees by depth in December (a), February (b), April (c) during the 2009/2010 growing season, Letaba, Kruger National Park, South Africa. Lower case letters indicate differences among depths for grasses. Upper case letters indicate differences among depths for trees. Asterisks indicates differences between grasses and trees at a depth. Significance was determined when P < 0.05.*
