# Supplementary material for: Modelling Water Uptake Provides a New Perspective on Grass and Tree Coexistence
Source: PLoS One. 2015 Dec 3;10(12):e0144300. doi: 10.1371/journal.pone.0144300 (PMC4669088; doi:10.1371/journal.pone.0144300)
Supplement: S5 Fig — Letaba, Kruger National Park, South Africa over the 2009–2010 growing season. predicted using tracer-derived estimates of root activity to parameterize the Hydrus 1D soil water model. The R2 values were 0.69, 0.59, 0.72, 0.80, 0.80 and 0.84 for panels a-f, respectively. A gravimetric water content of 0.12 is associated with a water potential of -2.5 MPa (i.e., plant unavailable water). (DOCX) [file pone.0144300.s005.docx]

***S5 Figure****. Predicted (blue circles) and observed (black circles) gravimetric soil water content (θ) at (a) 5, (b) 10, (c) 20, (d) 30, (e) 70 and (f) 0-90 cm. Letaba, Kruger National Park, South Africa over the 2009-2010 growing season. Values predicted using tracer-derived estimates of root activity to parameterize the Hydrus 1D soil water model. The R^2^ values were 0.69, 0.59, 0.72, 0.80, 0.80 and 0.84 for panels a-f, respectively. A gravimetric water content of 0.12 is associated with a water potential of -2.5 MPa (i.e., plant unavailable water).*
