# Supplementary material for: Modelling Water Uptake Provides a New Perspective on Grass and Tree Coexistence
Source: PLoS One. 2015 Dec 3;10(12):e0144300. doi: 10.1371/journal.pone.0144300 (PMC4669088; doi:10.1371/journal.pone.0144300)
Supplement: S6 Fig — Note that grass leaf area reported here was doubled when estimating transpiration. Across the growing season, grass leaf area was greater than tree leaf area (F3, 63 = 9.347, P = 0.002) and stomatal conductance was smaller for grasses than trees (F3,1596 = 22.75, P < 0.001). (DOCX) [file pone.0144300.s006.docx]

***S6 Figure****. (a) Leaf area and (b) mean stomatal conductance (g) for grasses and trees through the 2009-2010 growing season. Note that grass leaf area reported here was doubled when estimating transpiration. Across the growing season, grass leaf area was greater than tree leaf area (F_3, 63_ = 9.347, P = 0.002) and stomatal conductance was smaller for grasses than trees (F_3,1596_ = 22.75, P < 0.001).*
