# Supplementary material for: Modelling Water Uptake Provides a New Perspective on Grass and Tree Coexistence
Source: PLoS One. 2015 Dec 3;10(12):e0144300. doi: 10.1371/journal.pone.0144300 (PMC4669088; doi:10.1371/journal.pone.0144300)
Supplement: S1 Table — Values in a row followed by a different lower case letter are different at the 0.05 level. (DOCX) [file pone.0144300.s007.docx]

***S1 Table****. Mean δD excess values (‰) ± 1 SE in plant materials sampled from plots that had received tracer at the indicated soil depths (e.g., 5 cm) and time of year (e.g., December). Values in a row followed by a different lower case letter are different at the 0.05 level.*

| Plant type |  |  | Soil depth |  |  |
| --- | --- | --- | --- | --- | --- |
|  |  |  | Dec-09 |  |  |
|  | 5 cm | 10 cm | 20 cm | 30 cm | 70 cm |
| Grass | 130 ± 40a | NA* | 81 ± 11a | 124 ± 9a | 6 ± 1b |
| Tree | 18 ± 2b | NA* | 32 ± 5ab | 54 ± 5a | 16 ± 1b |
|  |  |  | Feb-10 |  |  |
| Grass | 306 ± 128a | 291 ± 76a | 177 ± 41a | 184 ± 26a | 13 ± 2b |
| Tree | 33 ± 5a | 62 ± 22a | 54 ± 5a | 38 ± 5a | 25 ± 2b |
|  |  |  | Apr-10 |  |  |
| Grass | 288 ± 107ab | 298 ±60a | 214 ± 42ab | 71 ± 12ab | 24 ± 3c |
| Tree | 43± 10ab | 56 ± 8a | 41 ± 9b | 67 ± 9a | 45 ± 3ab |

NA*=data not available
